# The 2024 NASEM Long COVID Definition as a Starting Point for Research

**DOI:** 10.1007/s11606-025-09916-6

**Published:** 2025-10-13

**Authors:** Andrea B. Troxel, Jerry A. Krishnan, Monica Verduzco-Gutierrez

**Affiliations:** 1 University of Illinois Chicago, Chicago, IL USA; 2 University of Texas at San Antonio, San Antonio, TX USA; 3 Department of Population Health, NYU Grossman School of Medicine, New York, NY USA

Five years after the identification of the novel respiratory virus SARS-CoV-2, the pathogenesis and treatment of neurocognitive impairment, exercise intolerance, post-exertional malaise, dysautonomia, and other chronic sequelae of SARS-CoV-2 infection remain poorly understood. Among the barriers to scientific progress is the lack of common terminology and definitions.^1^ Following a literature review, extensive deliberation involving stakeholders, and public meetings over 18 months, the National Academies of Science, Engineering, and Medicine (NASEM) recommended the adoption of the patient-coined term “Long COVID” and defined Long COVID as “an infection-associated chronic condition (IACC) that occurs after SARS-CoV-2 infection and is present for at least 3 months as a continuous, relapsing and remitting, or progressive disease state that affects one or more organ systems”.^2,3^ The committee provided justification for the definition’s elements in the full report,^2^ and recommended revisiting its definition as new information emerges. The inclusive definition of Long COVID reflects current evidence suggesting that Long COVID is heterogeneous in presentation and trajectory and recognizes the absence of clear causal pathways. The NASEM Committee prioritized inclusion (sensitivity) over specificity, acknowledging the nascency of research on this topic. In initial research, a more inclusive definition supports a range of studies examining the impact of infection with SARS-CoV-2 on different organ systems. Results of such research can be used to inform data-driven approaches and a more specific definition incorporating biomarkers linked to pathobiology. We acknowledge the challenge of establishing causality between symptoms and a previous SARS-CoV-2 infection, which arises from the immature state of science; further research about the pathobiology of Long COVID is underway. Importantly, the definition serves multiple purposes, including clinical diagnosis, research, public health surveillance and policy, and benefits provision and coverage. Here, we address its utility for research.

The 2024 NASEM definition of Long COVID has faced some skepticism about its utility from the research community. A common criticism is that the definition has limited specificity. Some are concerned that the definition fails to address the heterogeneity of Long COVID. Others would prefer that the definition incorporate semi-quantitative symptom-based algorithms in adults^4^ or children and adolescents^5^ that can differentiate individuals with versus without a previous SARS-CoV-2 infection.

Here, we offer responses to these critiques. Many of these issues were discussed at the National Institutes of Health (NIH) Researching COVID to Enhance Recovery–Treating Long COVID (RECOVER-TLC) Workshop in September 2024 to inform the planning of future clinical trials. We hope to identify opportunities to more effectively build on the 2024 NASEM definition of Long COVID and support the planning underway for the NIH RECOVER-TLC clinical trials consortium and other research efforts.

## SPECIFICITY

Critics argue that the 2024 NASEM Committee’s definition of Long COVID is excessively broad and risks including individuals without Long COVID, rendering it less useful for research. We contend, however, that the 2024 NASEM Long COVID definition provides a consensus-based starting point that is inclusive, as studies to date suggest that Long COVID includes more than 200 symptoms attributable to any organ system and may include exacerbations of pre-existing conditions immediately after SARS-CoV-2 infection or weeks to months later. There may also be longer-term IACCs (e.g., cancer) that are not yet apparent. Reflecting stakeholder input and acknowledging our incomplete understanding of IACCs, the NASEM Committee prioritized sensitivity (fewer false negative classifications) over specificity (fewer false positive classifications). The NASEM Committee encouraged investigators to build on the definition and use tailored study eligibility criteria to address their objectives. The RECOVER-TLC meeting identified common (e.g., post-exertional malaise) and uncommon but important (e.g., numbness) features of Long COVID that could be used to differentiate patient subgroups. The NASEM Committee encouraged researchers to provide a rationale for eligibility criteria, and to explain how they may affect the generalizability or interpretation of a study’s findings.

## HETEROGENEITY

The NASEM definition acknowledges that Long COVID involves heterogeneous presentations and trajectories. In research, such heterogeneity introduces variability and/or ambiguity in indicators of health status, disease pathogenesis, and response to interventions; this can lead to misdiagnosis and complicate our ability to evaluate study hypotheses. Restricting a study population using additional eligibility criteria can help to reduce heterogeneity, but may be insufficient. When considering clinical trials of potential treatments, platform protocols offer a promising approach to managing heterogeneity by balancing a broadly eligible population with specifically defined subgroups. This design has been used effectively in a number of settings, including breast cancer^6^ and acute COVID-19.^7^ Adaptive platform studies are designed to iteratively learn which intervention strategies are best, quickly screening out interventions unlikely to succeed and identifying those with a clinically meaningful effect. New interventions can be added rapidly as they are identified and ready for testing in a clinical trial. Multiple alternative interventions can be compared to a “standard of care” arm; new such standards may emerge from interim analysis results and evolving literature. The number of concurrent interventions might depend on the number of study sites and the overall pace of enrollment. The NASEM Long COVID definition aligns naturally with a platform design, beginning with a broad and commonly agreed definition of potentially eligible participants, and incorporating more specific requirements for individual interventions. Researchers studying Long COVID pathogenesis or epidemiology can similarly apply refinements to the broad definition to identify suitably targeted sub-populations.

## SEMI-QUANTITATIVE THRESHOLDS

Analyses of data collected from over 13,000 participants in the NIH RECOVER adult cohort study indicated that a threshold score of 11 or higher based on the presence or absence of eleven symptoms can differentiate individuals with versus without a previous SARS-CoV-2 infection.^4^ A report from the RECOVER pediatric cohort study in over 5,000 children and adolescents identified other semi-quantitative symptom thresholds (a score of 5.5 or higher from a list of ten symptoms in children 6 to 11 years, and a score of 5 or higher from among eight symptoms in adolescents 12 to 17 years).^5^ While appealing operationally, these threshold-based systems have not been validated in other populations and should not replace the broad definition serving multiple purposes and constituents. The studies’ authors acknowledged other limitations, including difficulty distinguishing the time-dependent nature of symptoms, misclassification of uninfected individuals due to decreased testing and milder acute disease, confounding by symptoms due to other undiagnosed conditions, and unclear representation of different variants and other selection and attrition biases in the cohort study. The NASEM definition does not preclude the use of such research indices, which could be applied to improve the ratio of true positives to false positives in a pool of patients eligible for a particular study. Alternatively, future studies may focus on a subset rather than all symptoms that contribute to research indices. In other words, eligibility criteria can and should be optimized for the particular purpose of each study.

## CONCLUSIONS

The 2024 NASEM Long COVID definition, designed to evolve with emerging evidence, provides a common framework for needed research. The current NASEM Long COVID definition also supports other pressing needs, including efforts to address equity and access to healthcare services and disability support. Research-relevant considerations, including specificity, heterogeneity, and concurrent use of symptom-based indices and thresholds, can be addressed effectively within this context. We encourage researchers to adopt the 2024 NASEM Long COVID definition as a useful starting point, and to articulate how modifications may affect the interpretation of research results.
